# Umbilical artery thrombosis diagnosed by fetal ultrasound

**DOI:** 10.1515/crpm-2024-0017

**Published:** 2024-09-12

**Authors:** Yushi Abe, Kazunori Ueno, Saki Tamura, Haruko Ariga, Jun Miyauchi, Hiroyuki Nakagawa

**Affiliations:** Department of Obstetrics and Gynecology, Saitama City Hospital, Saitama, Japan; Department of Obstetrics and Gynecology, Keio University School of Medicine, Tokyo, Japan; Department of Diagnostic Pathology, Saitama City Hospital, Saitama, Japan

**Keywords:** umbilical artery thrombosis, non-reassuring fetal status, fetal ultrasound

## Abstract

**Objectives:**

Umbilical artery thrombosis (UAT) is a rare and severe condition associated with grave perinatal outcomes, including intrauterine fetal death. This case report presents the case of a 38-year-old woman (gravida 3, para 1) of Japanese ethnicity, with a history of one spontaneous miscarriage, who conceived through micro-insemination and blastocyst transfer.

**Case presentation:**

Initial patient screening at 30 weeks and 6 days of gestation revealed normal fetal development, with two umbilical arteries and one umbilical vein. However, at 34 weeks and 5 days of gestation, we observed reduced fetal movements and the absence of accelerations on cardiotocography. Subsequent color Doppler examination revealed cessation of blood flow across a broad area in one umbilical artery and a strongly curved umbilical vein surrounding the blood flow of the other artery. These formed the ‘orange grab sign,’ suggestive of UAT. Evaluations of blood flow in other areas revealed unremarkable findings. We performed an emergency cesarean section owing to fetal distress. The mother and newborn were healthy and discharged as healthy. The 1-month check-up revealed no abnormalities in the child. Pathological examination of the umbilical cord revealed fibrin-based thrombus formation along the length of one artery, confirmed to be an umbilical artery.

**Conclusions:**

In the present case report, we presented the diagnostic challenges of UAT. Furthermore, we highlighted the need for timely intervention by comparing the number of umbilical vessels among previous ultrasound findings and verifying the presence of the ‘orange grab sign.’

## Introduction

Umbilical artery thrombosis (UAT), although infrequent, has serious implications for perinatal health, with outcomes potentially as grave as intrauterine fetal death. Its rarity underscores the importance of vigilant prenatal screening and early diagnostic interventions. The condition is characterized by the obstruction of blood flow due to a thrombus within an umbilical artery, which can lead to fetal compromise and a range of adverse perinatal outcomes.

The precise causes of UAT are not clearly defined; however, the potential associations include maternal clotting disorders, infections, and structural abnormalities of the umbilical cord. Clinically, UAT may be inadvertently discovered during routine ultrasound examinations or may present with concerning signs such as non-reassuring fetal heart patterns. Thus, accurate interpretation of ultrasound and Doppler images is imperative for diagnosis.

Differentiating UAT from congenital anomalies like single umbilical artery (SUA) is crucial, as each has distinct implications for the fetus. This differential diagnosis largely depends on ultrasound visualization of the umbilical cord’s vessel count and blood flow characteristics.

In this case report, we present the sonographic features of UAT, explore associated diagnostic challenges, especially in distinguishing it from SUA, and emphasize the urgency of appropriate management. By reviewing a UAT case, we aimed to reinforce the significance of meticulous prenatal ultrasound in improving perinatal outcomes.

## Case presentation

This report presents the case of a 38-year-old Japanese woman classified as gravida 3, para 1. The patient had no remarkable history or family medical history; however, the patient had previously experienced one spontaneous miscarriage. After unsuccessfully attempting the 6-month timing method for conception, the patient achieved pregnancy through micro-insemination and blastocyst transfer. The patient’s coagulation factors before pregnancy, including protein S, protein C, prothrombin time, and activated partial thromboplastin time, were within normal limits.

Fetal ultrasound screening at 30 weeks and 6 days of gestation revealed an estimated fetal weight of 1,505 g (31st percentile). We observed no signs suggestive of fetal structural abnormalities and noted two normal umbilical arteries and one umbilical vein.

**Figure 1:**
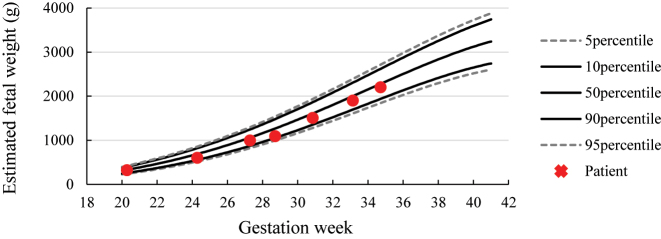
Fetal development curves. The dotted line represents the 5th and 95th percentiles, whereas the solid line represents the 10th, 50th, and 90th percentiles. Red crosses represent the weight of the child, as estimated by ultrasound examination.

**Figure 2:**
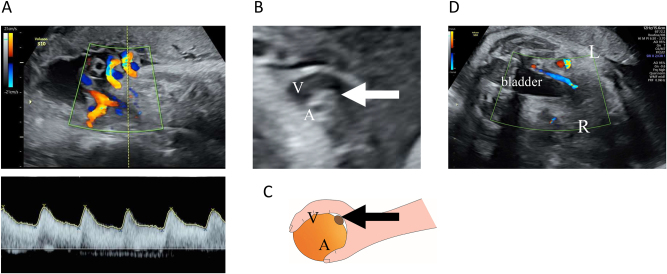
Ultrasound examination. (A) Transabdominal ultrasound images of the umbilical arteries and veins. Color flow mapping images of the umbilical cord blood flow. An opposing blood flow was seen in only one artery, indicating extensive disruption of blood flow in one umbilical artery. No abnormal findings were observed in the pulsed Doppler waveform for the umbilical artery with blood flow. (B and C) The ‘orange grab sign’ in B mode. A highly curved umbilical vein (V) surrounding an umbilical artery with blood flow (A) and an umbilical artery with an interrupted blood flow (arrow) was seen between them. (D) Horizontal section at the level of the fetal bladder. The right umbilical artery running on the right side of the bladder was observed to have an interrupted blood flow.

However, at 34 weeks and 5 days of gestation, the patient presented to the hospital after noticing reduced fetal movements. We confirmed decreased fetal movements via transabdominal ultrasound. The patient’s biophysical profile score was 4 out of 10, with positive findings for fetal tone and amniotic fluid pocket; however, there were no observed fetal breathing movements, fetal gross body movements, or reactive non-stress test. A fetal ultrasound revealed a fetal weight of 2,202 g (38th percentile). Figure 1 illustrates the estimated fetal weights at antenatal check-ups up to that point. Estimated fetal weight and percentiles were calculated based on the Japan Society of Ultrasonics in Medicine (JSUM) 2001 guidelines. Color flow mapping ultrasound showed blood flow arrest over a wide area in one umbilical artery; however, pulsed wave Doppler revealed no abnormal findings in the waveform of the umbilical artery with blood flow (Figure 2A). In addition, a strongly curved umbilical vein surrounding a blood-filled umbilical artery was seen, with small echoicity between these vessels. This finding was consistent with the ‘orange grab sign’ previously reported in UAT cases [1] (Figure 2B and C). Additionally, cessation of blood flow was observed in the right umbilical artery running along the right side of the fetal bladder (Figure 2D). Other blood flow assessments (e.g., in the left umbilical artery, middle cerebral artery, and venous duct assessments) did not reveal anomalies in waveform, flow velocity, or resistance. All ultrasound images were acquired using a Voluson S10 ultrasound machine and the RM6C transducer (GE Healthcare Japan, Tokyo, Japan). Based on these observations, we established a diagnosis of non-reassuring fetal status, and with the patient’s consent, performed an emergency cesarean section. The neonate’s Apgar scores were 8 and 9 after 1 and 5 min, respectively. Umbilical artery blood gas analysis performed immediately after birth showed a pH of 7.248 and a base excess of −3.8. The mother and neonate had good postoperative courses and were discharged as normal. We detected no abnormalities in the child at the 1-month check-up.

**Figure 3:**
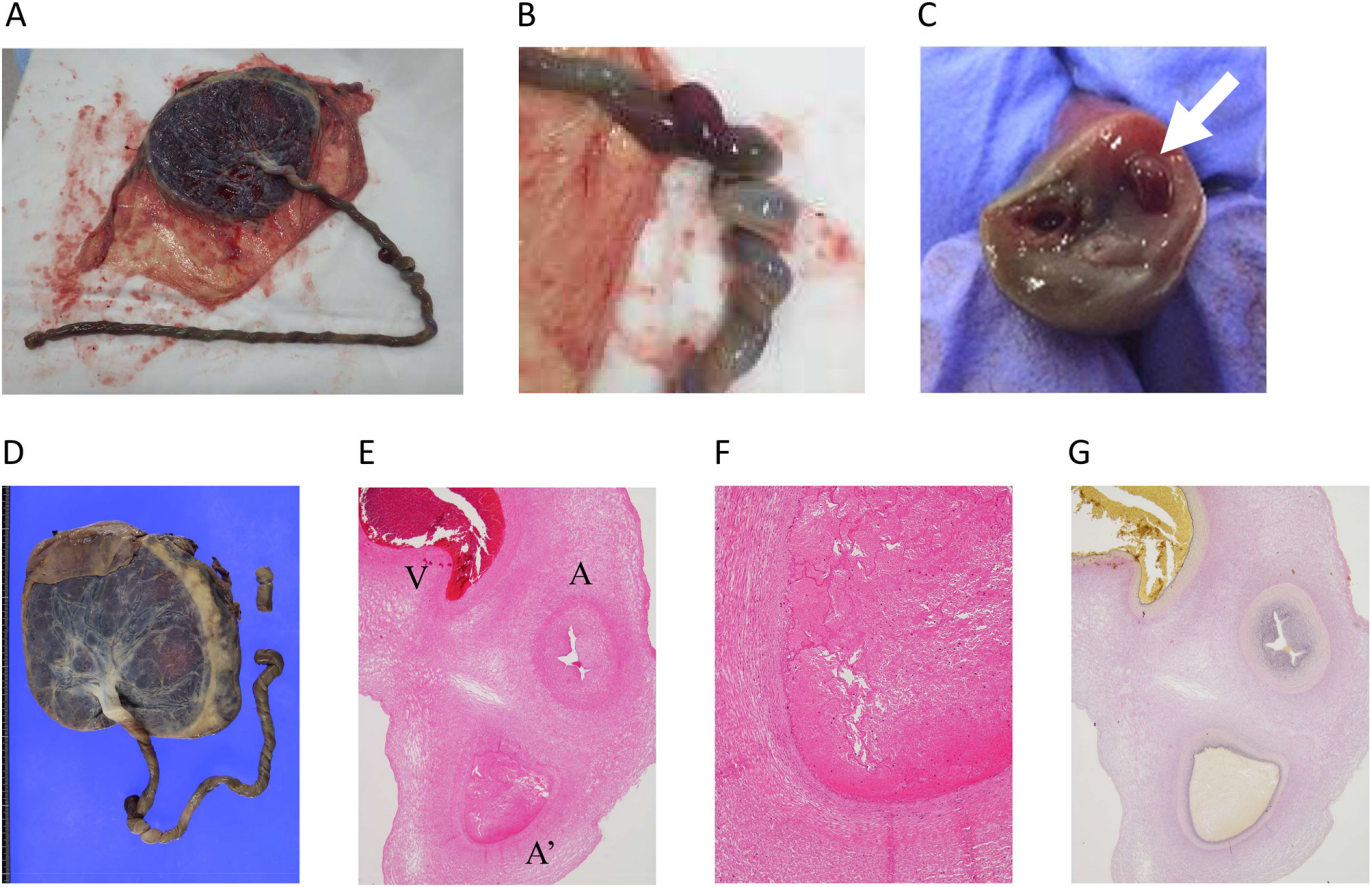
Macroscopic and histopathological finding. (A) Umbilical cord findings. A lengthy umbilical cord of 70 cm was observed. (B) Part of the umbilical cord was excessively coiled. (C) Three vascular vessels were present in a section of the umbilical cord, with a thrombus observed in the lumen of one of them (arrow). (D) Placenta after fixation. (E) Hematoxylin and eosin staining of a transverse section of the umbilical cord. The umbilical vein (V), umbilical artery (A), and occluded umbilical artery (A′) were observed. (F) Marked enlargement of the occluded umbilical artery in (B). No evidence of thrombus organization or necrosis of the arterial wall was observed. (G) Elastica van Gieson staining in serial sections. No elastic plates were observed in the occluded vessel.

The delivered umbilical cord of 70 cm showed localized hypercoiling (coiling index, 0.6; Figure 3A and B); the average umbilical cord length in Japan is 55.5 cm. We found three vascular vessels in a cord section, one with a thrombus in its lumen (Figure 3C). The umbilical cord was finely dissected for pathological analysis (Figure 3D), and all sections were evaluated following hematoxylin and eosin staining. The staining revealed a fibrin-dominated thrombus along nearly the entire length of one vessel (Figure 3E and F). Elastica van Gieson staining confirmed this vessel as an umbilical artery, owing to the absence of an elastic lamina around the vessel wall (Figure 3G). Furthermore, the intraluminal space of the umbilical artery near the placental insertion was completely obstructed by the thrombus, with no sign of thrombus organization or arterial wall necrosis, indicating that the thrombus had formed within a few days.

## Discussion

UAT is a rare disorder reported in approximately one in 1,300 deliveries, one in 1,000 postnatal pathological examinations of the umbilical cord and placenta, and one in 250 cases of high-risk pregnancy [2]. It is associated with severe complications, such as fetal asphyxia, intrauterine fetal death, and hypoxic-ischemic encephalopathy or cerebral palsy, resulting in poor perinatal outcomes. A retrospective study of 317 intrauterine fetal deaths suggested that UAT may have caused death in up to 10 % of cases [3].

Fetal ultrasound of a normal umbilical cord reveals one large vessel (umbilical vein) and two small vessels (umbilical arteries) in the cross-section. Color Doppler distinguishes these vessels using different colors and confirms their opposite blood flow directions. In UAT, blood flow ceases in one of the umbilical arteries, leaving only one vessel of each type (Figure 2A), and only one of the umbilical arteries is observed running alongside the fetal bladder (shown in Figure 2D). Besides, these findings are observed in single umbilical artery (SUA) cases, which can create diagnostic challenges [4]. SUA can result from congenital aplasia or early pregnancy atresia [5], both of which can be diagnosed by around 12 weeks of gestation [6]. In contrast, UAT typically occurs in the later stages of pregnancy. Essentially, SUA cases present with two visible vessels and blood flows from the outset, whereas UAT manifests as a reduction from three visible blood flows and vessels to two blood flows and three vessels. The presence of three vessels even after the onset of UAT is crucial in differentiating UAT from SUA. By comparing previous ultrasound findings of the umbilical cord, UAT can be differentiated from SUA.

The “orange grab sign” (Figure 2B and C), previously identified in umbilical cords with UAT [1], is considered characteristic of UAT and can be identified without comparing new and previous umbilical findings [7, 8]. Moreover, this sign reflects the presence of three vessels and is not observed when there are only two vessels.

Notably, most placentas have an anastomosis of the two umbilical arteries, a condition known as Hyrtl’s anastomosis. This anastomosis is thought to exist to equalize the pressure difference between the two umbilical arteries and ensure even blood flow to the placenta [9]. This can be understood from the fact that the Doppler waveforms of normal umbilical arteries show similar patterns in both arteries. Hyrtl’s anastomosis was first described by Hyrtl in 1870 after extensive examination of numerous placentas [10]. Recently, studies utilizing angiographic techniques have evaluated the anatomical structure of Hyrtl’s anastomosis and the regions of the placenta supplied by each umbilical artery. In these studies, Hyrtl’s anastomosis was absent in four of 67 cases (approximately 6 %). Furthermore, in placentas with narrow anastomoses, the symmetry of the placental areas supplied by each artery was higher than that in placentas with wider anastomoses, with the highest symmetry observed in placentas without anastomoses [11].

In Hyrtl’s anastomosis, even if the blood flow in one umbilical artery is obstructed, the anastomosis helps maintain some level of blood flow. Thus, if the umbilical artery with higher blood flow is impeded, a large Hyrtl’s anastomosis may prevent a sudden collapse of fetal circulation. Larger Hyrtl’s anastomoses are typically found in more asymmetrical placentas.

Conversely, if there is no Hyrtl’s anastomosis and blood flow in one artery is completely blocked, the remaining artery must handle twice the usual blood volume, leading to acute left heart overload, and only half of the placenta remains functional. Similar outcomes are expected if a thrombus progresses to occlude Hyrtl’s anastomosis. In the present case, we confirmed the presence of Hyrtl’s anastomosis and lack of obstruction of this site by the thrombus. However, we could not evaluate the size of Hyrtl’s anastomosis or the areas of the placenta supplied by each umbilical artery. In the future, it is necessary to accumulate evaluations of these parameters to better understand the hemodynamics in cases of UAT.

In conclusion, UAT is a rare condition that is profoundly associated with adverse perinatal outcomes, including intrauterine fetal death. Observing three vessels in the umbilical cord is crucial in differentiating UAT from SUA despite noting blood flow from only two of the vessels. The findings underscore the critical role of fetal ultrasound in the early detection and differentiation of UAT from similar conditions, such as single umbilical artery, particularly given its association with severe perinatal outcomes.
